# Editorial: Physical Activity “Enrichment”: A Joint Focus on Motor Competence, Hot and Cool Executive Functions

**DOI:** 10.3389/fpsyg.2021.658667

**Published:** 2021-03-09

**Authors:** Caterina Pesce, David F. Stodden, Kimberley D. Lakes

**Affiliations:** ^1^Department of Movement, Human and Health Sciences, University of Rome “Foro Italico,” Rome, Italy; ^2^Department of Physical Education, University of South Carolina, Columbia, SC, United States; ^3^Department of Psychiatry & Neuroscience, University of California, Riverside, Riverside, CA, United States

**Keywords:** exercise, sport, motor learning, cognition, memory, emotion, development, context

## Introduction

This topic has developed at the crossroads of three scientific paths: (1) physical activity (PA) and cognition research focused on the cognitive challenges involved in PA and motor skill acquisition (Pesce, 2012; Tomporowski and Pesce, 2019; Vazou et al., 2019); (2) developmental neuroscience research looking at PA as one of the means to aid cognitive—especially executive function (EF)—development within a joint physical, cognitive, and emotional framework (Diamond and Ling, 2016, 2020); (3) motor development research that flourished since the publication of Stodden et al. (2008) model and is moving toward a holistic perspective on the role of motor competence (MC) for physical, mental, and socio-emotional health. Thus, these contributions from exercise scientists, developmental neuroscientists, and motor developmentalists address the interconnectedness of PA, MC, cognitive and socio-emotional correlates or outcomes. Here, we integrate the individual contributions into a common framework to discuss current challenges and opportunities and posit implications for future research.

## Challenges and Opportunities Emerging From the Contributions to the Research Topic

Central to this Research Topic is the distinction and integration of “hot” and “cool” EF. Among the top-down neurocognitive control processes that fall under the umbrella term EF and are involved in goal-oriented behavior, the degree of affective and motivational salience have been used to distinguish “hot” and “cool” EFs. The latter are elicited under decontextualized and affectively neutral conditions, as when coping with tasks that require inhibiting interference, playing with ideas in mind, shifting flexibly between cognitive strategies. Instead, “hot” EF processes are performed in affectively salient contexts, as when coping with tasks that involve delaying a gratification/refraining from delay discounting, gambling, or making a risky decision with much to be gained or lost (Zelazo and Carlson, 2012). Studies addressing the MC-EF relation are mainly focused on “cool” EFs, while “hot” EFs are largely neglected (Lakes and Hoyt, 2004; Lakes et al., 2013; Pesce et al., 2020). The literature on “cool” EFs shows a nuanced pattern of selective associations between different facets of MC and EF constructs (van der Fels et al., 2015). Van der Veer et al. show that in preschool children, individual associations weaken when tested as unitary latent constructs of general MC and core EF. The non-linearity of early child development, as well as the “cool” nature of the EF tested, may be responsible for this dissociation. Indeed, Burns et al. found an association in older children between MC and on-task behavior that relies on “hot” EF. However, the strength of this association may be affected by individual and environmental factors, as it was observed in socially disadvantaged children.

The development of MC is “generally” universal and intransitive. It is also cumulative in that the development of skill variations builds a “coordinative pattern toolbox” that can be used at any time. There is no “correct or incorrect” movement *per se*, but rather variations of movement that can be chosen for a specific task goal that is embedded in a particular context. The notion that context-specific experiential learning inherently involves the development and utilization of EF (Pesce et al., 2019) is challenged by an Ecological Dynamics perspective, showing that new creative movement actions may emerge as a result of self-organization tendencies without “cool” EF involvement (Orth et al., 2019). This is the perspective used by Rudd, Pesce et al. to present learner-centered, non-linear pedagogies grounded on “explore-discovery-adapt.” The authors propose that “hot” rather than “cool” EFs may be more influential when learners search to utilize “affordances” offered by an enriched environment that has motivational salience and elicits emotional engagement.

PA enrichment and the contextual salience of motor skill learning are central to the contributions by Aadland et al. and Rudd, Crotti et al. which are protocol papers of large-scale interventions in Norway (ACTNOW) and the UK (SAMPLE-PE) with preschoolers and first graders. Both have a 2-fold holistic perspective: they bridge efficacy (i.e., child-level outcomes) and effectiveness research (i.e., evaluation of professional development and implementation processes) and target holistic development in motor, cognitive and socio-emotional domains.

PA enrichment includes both PA enriched with cognitive challenges and cognitive learning tasks enriched with PA. Vazou et al. pilot study tested the feasibility of an intervention with inherent coordinative and cognitive challenges and addresses factors that may indirectly contribute to linked benefits in the motor and EF domains, such as teacher support and motivational climate generated by peers. The other side of the coin is addressed by Damsgaard et al. and Amico and Schaefer. While Darmsgaard et al. used motor activities of low intensity (gestures) relevant to the learning subject, Amico and Schaefer used non-relevant PA tasks of higher intensity (running and dribbling). Both found that adding PA in the encoding phase improved learning in children regardless of its relevance to the learning subject (Mavilidi et al., 2018).

Tomporowski and Qazi address the issue of motor-cognitive dual task effects on memory within a broader, insightful intersection of theoretical perspectives that points to an interactive role of “hot” and “cool” EF. They suggest that bouts of exercise that provide a context for skill development, as those embedded into a learning task may benefit memory storage because they elicit positive affective experiences, engender goal-directed motivated action plans, and maintain mental engagement. However, Jung et al. discuss the limited transferability of dual task research findings to real life. They show the feasibility and cognitive advantage of dual-tasking in a virtual reality environment (Benzing and Schmidt, 2018) that mirrors real-life conditions, underlining the relevance of contextual salience to assess cognitive dual task effects.

The issue of ecological validity of the assessment tasks used to tap dual-task effects and EF is addressed also by Holfelder et al. to explain the absence of differences in “hot” EF between adolescent athletes, who are skilled performers in stable environmental situations (i.e., closed skill sports), or skilled in applying already-learned skills under situational uncertainty and time pressure (i.e., open skill sports). Both sport types may be expected to involve predictive and adaptive control with “hot” and “cool” EF components, yet in different ways. Holfelder et al. found some superiority among open skill athletes in “cool” EFs only, that emerges also as early as childhood, as shown by Moratal et al. The inability to find differences in “hot” EF raises the question of whether it is an issue of insufficient ecological validity of assessments, or insufficient stimulation by the PA and sport training.

“Hot” and “cool” EFs might display joint improvements following sensorimotor activities that are specifically tailored to activate emotional and cognitive states likely linked to brain regions involved in “hot” and “cool” EFs. This is the conclusion drawn by Leshem et al. in their review of neuroscientific evidence on the Quadrato Motor Training, a designed mindful movement training. Since mindful movements have displayed the highest consistency of evidence of EF benefits (Diamond and Ling, 2020), this may be a promising crossroads for research on MC, PA and “hot” and “cool” EFs.

## What Are the Implications for Assessment and Intervention?

### Enhancing Motor Competence and Executive Function Assessment

A more comprehensive assessment of MC that speaks to “real-world” performance and how it develops across time should include tasks that embody the integration of EFs (that have been developed or are developing) to trigger an optimal solution (i.e., pick the right movement solution from your toolbox) to accomplish the task goal. “Cool” EFs should be assessed by means of performances that are measured not only on a (milli)seconds time scale (Tomporowski et al., 2015) and “hot” EFs by means of responses to challenges in salient physically active or sport game contexts (Lakes, 2013; Pesce et al., 2020).

### Adopting a More Contextual Perspective to the Joint Promotion of Motor and Executive Function Development

We also need to attend more carefully to the intervention context. The traditional use of the terms “PA,” “exercise,” and “dose” by many authors in the public health arena and their linkage to enhanced EF speaks to neural development through mainly metabolic mechanisms that alter the brain structure and function (angiogenesis, neurogenesis). Unfortunately, this lacks an understanding of the contextual specificity of children's movement behaviors (i.e., learning to move and moving to learn) and the related task-specific synaptogenesis pathway in the brain regions involved in the learning task. Indeed, the nature and locus of neural plasticity seem specific to the unique features of the motor experience (Markham and Greenough, 2004; Adkins et al., 2006). Thus, all movement contexts for children should promote a dual focus that synergistically integrates the metabolic and learning (i.e., cognitively and motorically challenging) pathways responsible for angiogenesis, neurogenesis and synaptogenesis in the cortical and subcortical areas involved in EF and motor function to promote synergistic functioning and development (Tomporowski and Pesce, 2019).

To accomplish this, we need to reconceptualize the study and application of developmentally appropriate, holistic movement experiences that promote motor, “hot” and “cool” EF development. This will require a more contextual-specific perspective on emotional and cognitive development promotion, as it exists in the field of psychotherapy outcomes research (Wampold, 2001, 2015). Transitioning this model to PA-cognition research, interventions should directly challenge motoric functioning and EF (specific factors), while indirectly increasing contextual factors that support engagement in the activity (non-specific factors such as social support, joy, motivation), and indirectly decrease contextual factors that impair EF (non-specific factors such as stress, sadness, loneliness).

## Conclusions

Our Research Topic has highlighted the importance of advancing methods of assessment, both in MC and EF domains, and the design and implementation of targeted interventions, embracing a model that pays greater attention to the intervention context and to both specific and non-specific factors that concomitantly contribute to improved MC and “hot” and “cool” EF.

## Author Contributions

CP developed the initial proposal of the Research Topic. DS and KL provided relevant intellectual content to integrate and refine it. All authors of this Editorial equally contributed to its conception, development and writing, and agree to be accountable for the content of the work.

## Conflict of Interest

The authors declare that the research was conducted in the absence of any commercial or financial relationships that could be construed as a potential conflict of interest. The reviewer SWL declared a past co-authorship with one of the author DS.
